# Associations of breastfeeding duration and the total number of children breastfed with self-reported osteoarthritis in Korea women 50 years and older: a cross-sectional study

**DOI:** 10.4178/epih.e2023044

**Published:** 2023-04-13

**Authors:** Dajeong Ham, Sanghyuk Bae

**Affiliations:** 1 Department of Preventive Medicine, College of Medicine, The Catholic University of Korea, Seoul, Korea; 2 Environmental Health Center, The Catholic University of Korea, Seoul, Korea

**Keywords:** Osteoarthritis, Breastfeeding, Nutrition surveys

## Abstract

**OBJECTIVES:**

Osteoarthritis is the most common joint disease, with a higher prevalence among women than men. The present study aimed to examine the associations of breastfeeding duration and the total number of children breastfed with osteoarthritis in Korean women aged 50 years and older.

**METHODS:**

In this cross-sectional study, we used representative data from the Korea National Health and Nutrition Examination Survey, phases 5 through 7 (2010-2018). Our analysis included 10,102 women aged ≥50 years. Osteoarthritis experience was defined as whether a physician had ever diagnosed osteoarthritis. Breastfeeding duration was categorized as 1-6 months, 7-24 months, and ≥25 months. The total number of children breastfed was categorized as 1-2, 3-4, and ≥5. The covariates were health behavior characteristics and risks of diseases (smoking, drinking, physical activity, body mass index, diabetes, hypertension, oral contraceptive use, and menopause) as well as socioeconomic characteristics (income, educational level, and occupation). A multiple logistic regression model was used to investigate associations between osteoarthritis and aspects of breastfeeding experience.

**RESULTS:**

Compared to the non-breastfeeding group, the breastfeeding group had an odds ratio (OR) of 1.55 (95% confidence interval [CI], 1.18 to 2.03) for osteoarthritis. Those who reported breastfeeding for >25 months had an OR of 1.56 (95% CI, 1.19 to 2.06).

**CONCLUSIONS:**

The advantages of breastfeeding are already well known, but the present study suggests that women who breastfeed children for a longer time may have a higher risk of osteoarthritis after middle age.

## GRAPHICAL ABSTRACT

[Fig f2-epih-45-e2023044]

## INTRODUCTION

Osteoarthritis is a condition in which the cartilage present in the joints between bones is worn out and the adjacent bone surfaces are exposed, causing inflammation of the membrane surrounding the joint. Osteoarthritis is the most common joint disease [1,2]. Osteoarthritis affects 31% of Korean adults aged 65 years and older [3], and globally, 9.6% of men and 18.0% of women over 60 years of age are affected [2]. As the total number of elderly people increases worldwide, the proportion of affected individuals is expected to increase, and the resulting economic burden for individuals and society is also expected to be high [4,5]. Osteoarthritis has many risk factors, such as age, gender, heredity, body mass index (BMI), smoking, alcohol use, nutrition, hormone levels, bone density, trauma, physical activity, muscle strength, and occupation [5].

The prevalence of osteoarthritis is higher in women than in men [6,7]. Women experience various hormonal changes due to pregnancy, childbirth, and menopause [8,9], including estrogen deficiency, which may cause bone loss [10-13]. One risk factor affecting hormone levels in women is breastfeeding. Breastfeeding has long-term and short-term benefits for both mother and child [14]. It lowers the maternal risks of obesity, type 1 and 2 diabetes, hypertension, cardiovascular disease, hyperlipidemia, and cancer [14,15]. Breastfeeding also has many psychological benefits, such as a lower chance of maternal depression [16]. For children, it helps their cognitive and social development [16]. However, breastfeeding may also have negative effects such as pain [17], sexual dysfunction [18], and viral infections [19,20]. Recent studies have investigated the risk of subsequent osteoarthritis [21-23].

Many studies have examined the association between breastfeeding and osteoporosis risk [24-26]. However, evidence for an association between breastfeeding and osteoarthritis is inconsistent, and the association between the number of children breastfed and osteoarthritis has not been studied previously. In an earlier study examining the association between osteoarthritis and menopause, estrogen deficiency after menopause worsened osteoarthritis [26-28]. Additionally, several studies have shown that postmenopausal estrogen replacement therapy can prevent osteoarthritis [26-28]. An earlier study on the association of osteoarthritis with childbirth and lactation found that women aged ≥ 50 years who had breastfed a child for at least one month had a higher risk of developing osteoarthritis than women who had breastfed for less than one month [21]. In contrast, a recent study reported that the number of children and duration of breastfeeding were not associated significantly with osteoarthritis [23]. The inconsistency in results between studies may be due to differences in survey methods, sample selection, number of samples, and analytical methods.

The present study aimed to analyze the associations of breastfeeding duration and the total number of children breastfed with the prevalence of osteoarthritis in a nationally representative sample of Korea National Health and Nutrition Examination Survey (KNHANES) data, using all available data on breastfeeding duration and the total number of children.

## MATERIALS AND METHODS

### Study population

KNHANES is a nationwide health and nutrition survey conducted by the Korea Disease Control and Prevention Agency. This cross-sectional survey includes approximately 10,000 individuals each year and collects information on socioeconomic conditions, health-related behaviors, quality of life, medical use, human measurements, and biochemical and clinical profiles with three component surveys: health interview, health examination, and nutrition survey. The present study used KNHANES data conducted during the fifth (2010-2012), sixth (2013-2015), and seventh (2016-2018) phases [29].

**Figure 1.**
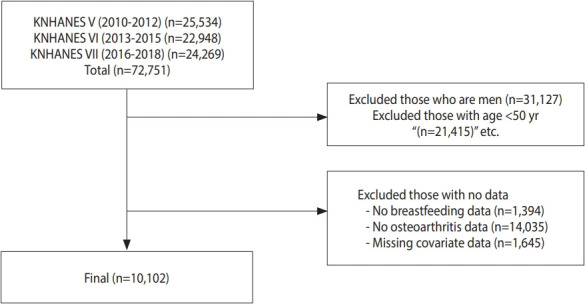
Flowchart showing the participant selection process. KNHANES, Korea National Health and Nutrition Examination Survey.

The total number of participants in these phases was 72,751. After excluding men, individuals aged < 50 years, and those who had no breastfeeding data, no osteoarthritis data, or missing covariate data, a total of 10,102 women were included in the analyses (Figure 1).

### Outcome measurement

Participants with osteoarthritis were defined as those who answered “yes” to the question “Have you ever been diagnosed with osteoarthritis?” in the health questionnaire, or those diagnosed by a physician during the survey based on radiological examination of any joint.

### Exposure assessment

Those with breastfeeding experience were defined as those who answered “yes” to the question “Have you ever breastfed for at least a month?” We also used the answers to the questions “What is the total number of children breastfed?” and “How long was the total period of breastfeeding?” Breastfeeding duration was categorized as 1-6 months, 7-24 months, and ≥ 25 months based on the World Health Organization-recommended periods [30]. The total number of children breastfed was categorized as 1-2, 3-4, and ≥ 5.

### Covariates

Potential confounding factors were selected a priori from the literature [31,32]. Covariates were also derived from answers to the questionnaire. The socio-demographic covariates were age, income (lowest, medium-low, medium-high, and highest), educational level (elementary, middle school, high school and college), and occupation (professionals and related workers, office workers, service and sales workers, agricultural, forestry and fishery trades, technical workers and machinery operation and assembly workers, manual labor jobs, and unemployed). The health and behavior covariates were defined as smoking status (non-smoking, ex-smoker, and current smoker), physical activity (yes or no), drinking experience (yes or no), BMI (underweight: < 18.5; normal: ≥ 18.5 and < 23.0; pre-obesity: ≥ 23.0 and < 25.0; stage 1 obesity: ≥ 25.0 and < 30.0; and stage 2-3 obesity ≥ 30.0 kg/m^2^), hypertension (hypertension, pre-hypertension, and normal), diabetes (normal, impaired fasting glucose, and diabetes). Experience with oral contraceptives was measured on the health questionnaire by “Have you taken oral contraceptives for at least 1 month?” (yes or no), and those who answered “natural menopause” or “artificial menopause” were classified as menopausal, whereas those who answered “menstrual period,” “pregnancy,” or “lactating after childbirth” were classified as non-menopausal. Parity was measured from the number of total pregnancies from the health questionnaire.

### Statistical analysis

KNHANES uses stratified cluster sampling with weights. Therefore, the statistical analysis was conducted using composite sample data that accounted for sampling weights. For categorical variables, data were presented as frequencies and percentages, and the chi-square test was used for comparisons. For continuous variables, data were presented as mean and standard deviation, and analysis of variance was used for comparisons. In the main analysis, a multiple logistic regression model was used to examine the associations of breastfeeding duration and the number of breastfed children with self-reported osteoarthritis. We adjusted for potential confounders: in model 1, we adjusted only for age; in model 2, we additionally adjusted for health and behavior covariates (smoking experience, drinking experience, physical activity, BMI, diabetes, hypertension, use of oral contraceptives, and menopause); and in model 3, we additionally adjusted for socioeconomical covariates (income level, educational level, and occupation). Due to the high prevalence of osteoarthritis in the elderly, subgroup analyses were conducted and stratified by age in years: 50s, 60s, and 70s or older. Since data on osteoarthritis prevalence were collected by 2 methods during part of KNHANES (fifth and sixth phases, conducted in 2010-2013)−namely, a self-administered questionnaire and radiological findings—a sensitivity analysis was conducted to investigate whether these 2 methods yielded different results. Odds ratios (ORs) and 95% confidence intervals (CIs) were presented. The Cochran-Armitage trend test was used to analyze trends in breastfeeding duration and the number of breastfed children. Multicollinearity was tested by the variance inflation factor (VIF).

### Ethics statement

The protocol of the present study was approved by the Institutional Review Board at The Catholic University of Korea (MC20ZASI0133).

## RESULTS

Table 1 shows the general characteristics of the participants. Women with breastfeeding experience were generally older and reported lower income and educational levels than those who reported no breastfeeding experience. Those with longer breastfeeding periods were more likely to report lower educational levels, diabetes, and high blood pressure.

Table 2 shows the association between breastfeeding duration and osteoarthritis. After adjusting for potential confounders, the group with breastfeeding experience had an OR of 1.55 (95% CI, 1.18 to 2.03) compared to the non-breastfeeding group. The associations were similar according to the breastfeeding duration (p for trend < 0.001). The number of breastfed children was also associated significantly with osteoarthritis (p for trend < 0.001). Compared to the non-breastfeeding group, the group that had breastfed 3 or 4 children had an OR of 1.37 (95% CI, 1.10 to 1.71), and the group that had breastfed 5 or more children had an OR of 1.55 (95% CI, 1.18 to 2.03) (Table 3).

Age-stratified analysis showed no significant association among women in their 50s. However, for those in their 60s or ≥ 70 years of age, the associations were significant for breastfeeding experience lasting 1-6 months (Table 4). Among those ≥ 50 years, the OR was 1.53 (95% CI, 1.07 to 2.21) for those who had breastfed 3 or 4 children. For participants who had breastfed 5 or more children, those in their 60s had an OR of 2.04 (95% CI, 1.24 to 3.35), and those aged 70s and older had an OR of 1.96 (95% CI, 1.19 to 3.23) (Table 5).

Supplementary Material 1 shows the VIF from the regression analysis. All variables had a VIF < 10. Results of subgroup analyses with radiologically diagnosed osteoarthritis showed a similar pattern (Supplementary Materials 2-5).

## DISCUSSION

In the present study, breastfeeding experience was associated with a higher prevalence of osteoarthritis among women aged ≥ 50 years in the nationally representative survey in Korea. Compared to the non-breastfeeding group, the breastfeeding group had an OR of 1.55 (95% CI, 1.18 to 2.03) for osteoarthritis. After adjusting for the number of children breastfed, those who reported a breastfeeding duration of > 25 months showed a significant association with osteoarthritis (OR, 1.56; 95% CI, 1.19 to 2.06). That is, participants were more likely to have osteoarthritis the longer they had breastfed, regardless of the number of children. This finding is important because the number of children breastfed was also significantly associated with osteoarthritis (OR, 1.37; 95% CI, 1.10 to 1.71 for 3-4 children and OR, 1.55; 95% CI, 1.18 to 2.03 for 5 or more children, respectively).

American women over 50 years of age with a history of breastfeeding were found to have a higher risk of developing osteoarthritis than women who did not breastfeed (OR, 1.21; 95% CI, 1.05 to 1.40) [21]. Among Korean women over 50 years of age, the prevalence of knee osteoarthritis diagnosed radiologically among those who had breastfed for 25-48 months was higher than that among non-breastfeeding women (OR, 2.30; 95% CI, 1.09 to 4.86); for those with a breastfeeding duration of more than 48 months, the prevalence of knee osteoarthritis was higher than that in non-breastfeeding women (OR, 2.17; 95% CI, 1.01 to 4.64) [22]. In a study using Women’s Health Initiative data, among postmenopausal women aged ≥ 50 years, those who had breastfed for 1-3 months (OR, 1.026; 95% CI, 1.017 to 1.034) or 4-6 months (OR, 1.015; 95% CI, 1.006 to 1.025) were more likely to self-report osteoarthritis than those who had never breastfed [23]. We found no studies examining the association between the number of children breastfed and osteoarthritis. The present study provides additional evidence on the association between breastfeeding and self-reported osteoarthritis in any joint.

Women hormones may explain the underlying mechanism supporting the present study’s findings. Estrogen increases during pregnancy and temporarily decreases postpartum, including during lactation [8,9], with a further decrease after menopause. Estrogen deficiency can affect collagen synthesis, bone density, and maintenance of chondrocytes [10,11,27]. Bone loss through estrogen deficiency is due to the complex interaction of hormones and cytokines that interfere with the bone remodeling process. Estrogen inhibits the activity of T cells by inhibiting interleukin-7 and interferon-γ production in the bone marrow, thymus, and peripheral lymphoid organs, and it acts to suppress the production of tumor necrosis factor, a factor that also stimulates osteoclast production. However, estrogen deficiency results in an imbalance in the production-inhibition system of these factors, exacerbating bone loss [12]. In fact, according to a study that confirmed the risk of developing osteoarthritis by measuring the concentration of major estrogen metabolites through a urine test before osteoarthritis was assessed and diagnosed, the risk of osteoarthritis of the knee is high if the concentration of estrogen metabolites is low [33].

Breastfeeding has many advantages and is recommended. It reduces the risks of developing breast cancer, ovarian cancer, and type 1 and 2 diabetes, and it extends the duration of postpartum infertility [14-16,34]. However, the current study shows that women who have breastfed for a long time and have breastfed many children may be at higher risk for osteoarthritis. In other words, although breastfeeding is not a direct cause of osteoarthritis, extended periods of lactation may be more likely to lead to osteoarthritis, regardless of the specific pathway. Therefore, caution is needed regarding osteoarthritis management (weight control, early screening, etc.) in this population.

The present study has several limitations. First, the cross-sectional study design prevents inferring causality between breastfeeding and osteoarthritis. The results of this study present a noncausal association, which may be useful for identifying individuals at higher risk. Second, because a questionnaire was used to assess exposure, recall bias is possible. In particular, for older participants, more time is likely to have passed between their experiences of breastfeeding and the survey. Although we observed similar associations in analyses stratified by age, recall bias remains possible within strata. Among the variables used for analysis, osteoarthritis is usually diagnosed by comprehensively considering radiological findings, physical examination, and past history. Although a recent study using KNHANES noted minimal overlap between radiographically diagnosed and self-reported osteoarthritis [35], KNHANES has a quality-control program to manage measurement errors such as estimation and recall problems and interviewer mistakes [36]. Furthermore, questionnaires are widely recognized in health research as a useful tool to gather information on behaviors such as smoking and exercise. Third, osteoarthritis is a disease that occurs in specific parts of the body, such as knees, hips, spine, and fingers. However, because the KNHANES questionnaire for self-reported osteoarthritis does not collect data on which joint(s) may be affected, this aspect could not be analyzed. Fourth, breastfeeding may involve varying amounts and methods, such as single feeding and mixed feeding. Because KNHANES does not contain the relevant data, we could not analyze the effects of different breastfeeding methods.

The advantages of breastfeeding are well known, but the present study suggests that women who have breastfed for longer periods may have a higher risk of developing osteoarthritis after middle age. An individual’s breastfeeding history may be used to evaluate the risk of osteoarthritis.

## Figures and Tables

**Figure f2-epih-45-e2023044:**
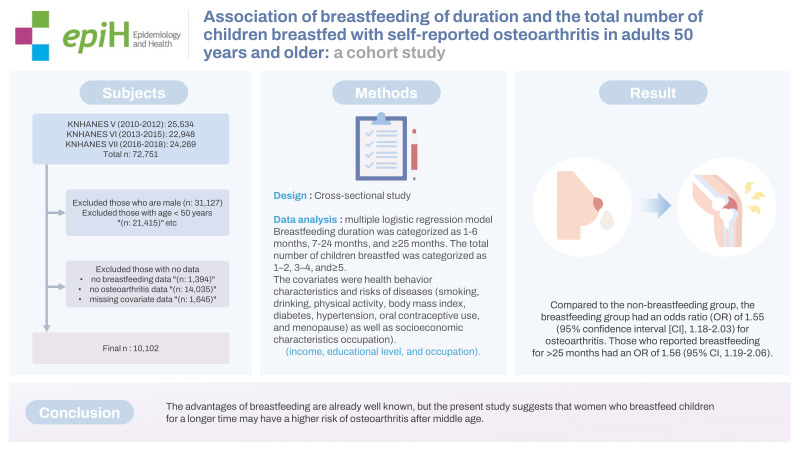


**Table 1.** General characteristics of participants according to breastfeeding duration

Characteristics		All (n=10,102)	Breastfeeding experience (mo)				p value^1^
			None (n=965)	1-6 (n=754)	7-24 (n=3,120)	≥25 (n=5,263)	
Age (yr)		62.7±0.1	57.7±0.3	56.6±0.2	58.7±0.1	67.8±0.2	<0.001
Income							
	Lowest	2,354 (23.3)	188 (2.1)	134 (1.4)	671 (7.0)	1,361 (12.6)	<0.001
	Medium-low	2,527 (25.1)	230 (2.7)	173 (1.8)	749 (8.2)	1,375 (12.3)	
	Medium-high	2,571 (25.4)	251 (2.7)	171 (2.0)	840 (9.0)	1,309 (11.5)	
	Highest	2,650 (26.1)	296 (3.1)	276 (2.9)	860 (9.0)	1,218 (10.9)	
Educational level							
	Elementary	5,098 (46.2)	225 (2.1)	118 (1.2)	939 (9.4)	2,342 (80.8)	<0.001
	Middle school	1,674 (17.3)	114 (1.4)	106 (1.1)	698 (7.5)	3,816 (33.4)	
	High school	2,302 (25.3)	343 (4.0)	304 (3.4)	1,106 (12.4)	756 (7.2)	
	College	1,028 (10.9)	283 (3.2)	226 (2.5)	377 (3.8)	549 (5.4)	
Occupation							<0.001
	Managers & specialists	345 (3.7)	84 (0.9)	83 (0.9)	126 (1.3)	52 (0.4)	
	Clerks	256 (2.8)	63 (0.6)	45 (0.5)	103 (1.2)	45 (0.4)	
	Sales workers	1,462 (16.2)	153 (1.7)	142 (1.6)	656 (7.5)	511 (5.2)	
	Agricultural, forestry, & fishery workers	690 (5.6)	19 (0.1)	10 (0.1)	114 (1.0)	547 (4.3)	
	Engineers & technicians	260 (2.7)	23 (0.2)	27 (0.3)	127 (1.3)	83 (0.7)	
	Manual laborers	1,378 (14.2)	129 (1.4)	79 (0.9)	436 (4.8)	734 (6.9)	
	None	5,711 (54.4)	494 (5.4)	368 (3.8)	1,558 (16.0)	3,291 (29.1)	
Smoking status							<0.001
	Non-smoking	9,572 (94.4)	875 (9.8)	697 (7.6)	2,960 (31.5)	5,040 (45.3)	
	Ex-smoker	231 (2.2)	47 (0.5)	24 (0.2)	63 (0.6)	97 (0.8)	
	Current smoker	299 (3.2)	43 (0.4)	33 (0.4)	97 (1.0)	126 (1.2)	
Physical activity							<0.001
	Yes	8,196 (80.5)	738 (8.2)	550 (6.0)	2,410 (25.4)	4,498 (40.7)	
	No	1,906 (19.4)	227 (2.5)	204 (2.2)	710 (7.8)	765 (6.7)	
Drinking experience							<0.001
	Yes	2,810 (25.9)	228 (2.4)	126 (1.2)	651 (6.2)	1,805 (15.9)	
	Not obese	7,292 (74.1)	737 (8.3)	628 (7.0)	2,469 (27.0)	3,458 (31.5)	
Body mass index							<0.001
	Underweight	175 (1.8)	32 (0.3)	19 (0.2)	50 (0.5)	74 (0.6)	
	Normal	3,467 (35.3)	427 (4.9)	349 (3.9)	1,175 (12.7)	1,516 (13.7)	
	Pre-obesity	2,568 (24.8)	242 (2.6)	178 (1.8)	801 (8.4)	1,347 (11.8)	
	Stage 1 obesity	3,334 (32.6)	226 (2.4)	177 (1.9)	939 (10.0)	1,992 (18.1)	
	Stage 2-3 obesity	558 (5.3)	38 (0.4)	31 (0.3)	155 (1.6)	334 (3.0)	
Diabetes							<0.001
	Normal	5,793 (57.7)	590 (6.6)	509 (5.5)	1,962 (21.2)	2,732 (24.2)	
	IFG	2,597 (25.9)	253 (2.7)	164 (1.8)	779 (8.5)	1,401 (12.7)	
	Diabetes	1,712 (16.2)	122 (1.4)	81 (0.9)	379 (3.5)	1,130 (10.3)	
Hypertension							<0.001
	Normal	2,850 (30.9)	376 (4.5)	325 (3.6)	1,117 (12.6)	1,032 (10.0)	
	Pre-hypertension	2,304 (23.2)	237 (2.6)	199 (2.2)	778 (8.4)	1,090 (9.8)	
	Hypertension	4,948 (45.7)	352 (3.5)	230 (2.4)	1,225 (12.2)	3,141 (27.5)	
Oral contraceptive experience							<0.001
	Yes	2,291 (21.7)	140 (1.6)	116 (1.1)	633 (6.4)	1,402 (12.4)	
	No	7,811 (78.2)	825 (9.1)	638 (7.1)	2,487 (26.8)	3,861 (35.0)	
Menopause status							<0.001
	Yes	612 (7.7)	138 (1.7)	124 (1.6)	248 (3.1)	102 (1.2)	
	No	9,490 (92.2)	827 (9.0)	630 (6.7)	2,872 (30.2)	5,161 (46.2)	
Parity		4.50±0.02	3.40±0.05	3.60±0.07	3.80±0.03	3.50±0.03	

Values are presented as mean±standard deviation or number (%). IFG, impaired fasting glucose.

1 Using the chi-square test for categorical variables and analysis of variance for continuous variables.

**Table 2.** Association between breastfeeding duration and osteoarthritis among women ≥50 years^1^

Variables		Unadjusted	Model 1	Model 2	Model 3
Breastfeeding duration					
	None	1.00 (reference)	1.00 (reference)	1.00 (reference)	1.00 (reference)
	Any duration	2.30 (1.92, 2.77)	1.62 (1.34, 1.96)	2.04 (1.56, 2.65)	1.55 (1.18, 2.03)
Breastfeeding experience (mo)					
	None	1.00 (reference)	1.00 (reference)	1.00 (reference)	1.00 (reference)
	1-6	0.96 (0.73, 1.25)	1.03 (0.79, 1.35)	1.45 (1.03, 2.05)	1.41 (1.00, 2.00)
	7-24	1.49 (1.22, 1.82)	1.44 (1.18, 1.75)	1.85 (1.37, 2.48)	1.55 (1.15, 2.10)
	≥25	3.44 (2.85, 4.17)	2.04 (1.66, 2.51)	2.11 (1.61, 2.75)	1.56 (1.19, 2.06)
	p for trend	<0.001	<0.001	<0.001	<0.001

Values are presented as odds ratio (95% confidence interval).

1 Model 1 was adjusted for age; Model 2 was adjusted for age, body mass index, smoking status, drinking experience, physical activity, diabetes, hypertension, use of oral contraceptives, menopause status, total number of children breastfed, and parity; Model 3 was adjusted for age, income, educational level, occupation, body mass index, smoking status, drinking experience, physical activity, diabetes, hypertension, use of oral contraceptives, menopause status, total number of children breastfed, and parity.

**Table 3.** Association between total number of children breastfed and osteoarthritis among women ≥50 years^1^

Total no. of children breastfed	Unadjusted	Model 1	Model 2	Model 3
None	1.00 (reference)	1.00 (reference)	1.00 (reference)	1.00 (reference)
1-2	1.45 (1.20, 1.76)	1.39 (1.14, 1.70)	1.26 (1.04, 1.53)	1.13 (0.92, 1.38)
3-4	3.26 (2.68, 3.96)	2.10 (1.70, 2.58)	1.75 (1.41, 2.17)	1.37 (1.10, 1.71)
≥5	5.58 (4.62, 7.32)	2.50 (1.94, 3.23)	2.04 (1.56, 2.66)	1.55 (1.18, 2.03)
p for trend	<0.001	<0.001	<0.001	<0.001

Values are presented as odds ratio (95% confidence interval).

1 Model 1 was adjusted for age; Model 2 was adjusted for age, body mass index, smoking status, drinking experience, physical activity, diabetes, hypertension, use of oral contraceptives, menopause status, and parity; Model 3 was adjusted for age, income, educational level, occupation, body mass index, smoking status, drinking experience, physical activity, diabetes, hypertension, use of oral contraceptives, menopause status, and parity.

**Table 4.** Association between breastfeeding duration and osteoarthritis, stratified by age^1^

Variables	Age (yr)		
	50-59 (n=3,693)	60-69 (n=3,406)	≥70 (n=3,003)
Breastfeeding duration			
None	1.00 (reference)	1.00 (reference)	1.00 (reference)
Any duration	0.94 (0.86, 1.04)	2.04 (1.24, 3.35)	1.96 (1.19, 3.23)
Breastfeeding experience (mo)			
None	1.00 (reference)	1.00 (reference)	1.00 (reference)
1-6	1.64 (0.59, 4.59)	2.35 (1.27, 4.35)	2.39 (1.02, 5.59)
7-24	1.85 (0.68, 4.99)	2.22 (1.30, 3.80)	2.30 (1.25, 4.23)
≥25	2.20 (0.82, 5.88)	2.01 (1.22, 3.31)	1.94 (1.18, 3.30)
p for trend	<0.001	<0.001	<0.001

Values are presented as odds ratio (95% confidence interval).

1 Adjusted for income, education level, occupation, body mass index, smoking status, drinking experience, physical activity, diabetes, hypertension, use of oral contraceptives, menopause status, total number of children breastfed, and parity.

**Table 5.** Association between total number of children breastfed and osteoarthritis, stratified by age^1^

Total no. of children breastfed	Age (yr)		
	50-59 (n=3,693)	60-69 (n=3,406)	≥70 (n=3,003)
None	1.00 (reference)	1.00 (reference)	1.00 (reference)
1-2	1.23 (0.92, 1.66)	0.75 (0.53, 1.06)	1.47 (0.89, 2.40)
3-4	1.53 (1.07, 2.21)	0.91 (0.63, 1.31)	1.68 (1.03, 2.72)
≥5	2.14 (0.80, 5.73)	2.04 (1.24, 3.35)	1.96 (1.19, 3.23)
p for trend	<0.001	<0.001	<0.001

Values are presented as odds ratio (95% confidence interval).

1 Adjusted for income, education level, occupation, body mass index, smoking status, drinking experience, physical activity, diabetes, hypertension, use of oral contraceptives, menopause status, and parity.

## SUPPLEMENTARY MATERIALS

Supplementary Material 1. Result of Variance Inflation Factors of regression analysis(model 3)

Supplementary Material 2 Association between breastfeeding duration with radiologically diagnosed osteoarthritis among aged over 50 years, 2010-2013

Supplementary Material 3 Association between total number of children breastfed with radiologically diagnosed osteoarthritis among aged over 50 years, 2010-2013

Supplementary Material 4 Association between breastfeeding duration and with radiologically diagnosed osteoarthritis stratified by age group, 2010-2013

Supplementary Material 5 Association between total number of children breastfed with radiologically diagnosed osteoarthritis stratified by age group, 2010-2013
