# Supplementary material for: Associations of breastfeeding duration and the total number of children breastfed with self-reported osteoarthritis in Korea women 50 years and older: a cross-sectional study
Source: Epidemiol Health. 2023 Apr 13;45:e2023044. doi: 10.4178/epih.e2023044 (PMC10396802; doi:10.4178/epih.e2023044)
Supplement: Supplementary Material 1. — Result of Variance Inflation Factors of regression analysis(model 3) [file epih-45-e2023044-Supplementary-1.docx]

**Supplementary Material File**

Results of subgroup analyses with radiologically diagnosed osteoarthritis.

Because in part of the KNHANES (5th, conducted during 2010-2012; 6th, conducted in 2013) osteoarthritis was collected by two diagnostic methods, namely, a self-question-answer questionnaire and radiological findings, subgroup analysis was additionally conducted to investigate whether there was a difference in the results between the two diagnostic methods

**Supplementary Material 1.** **Result of Variance Inflation Factors of regression analysis(model 3)**

| Variable | VIF | Tolerance(1/VIF) |
| --- | --- | --- |
| Age | 0.21 | 4.72 |
| Income | 0.89 | 1.12 |
| Education | 0.41 | 2.42 |
| Occupation | 0.86 | 1.16 |
| Obesity | 0.85 | 1.17 |
| Hypertension | 0.62 | 1.60 |
| Diabetes | 0.84 | 1.19 |
| Physical activity | 0.96 | 1.04 |
| Smoking status | 0.96 | 1.04 |
| Drinking experience | 0.88 | 1.14 |
| Oral contraceptives pill experience | 0.97 | 1.03 |
| Total number of children breastfed | 0.34 | 2.97 |
| Menopause status | 0.33 | 3.01 |
| Parity | 0.93 | 1.08 |
| VIF, Variance Inflation Factors. |  |  |
