# Supplementary material for: Associations of breastfeeding duration and the total number of children breastfed with self-reported osteoarthritis in Korea women 50 years and older: a cross-sectional study
Source: Epidemiol Health. 2023 Apr 13;45:e2023044. doi: 10.4178/epih.e2023044 (PMC10396802; doi:10.4178/epih.e2023044)
Supplement: Supplementary Material 2 — Association between breastfeeding duration with radiologically diagnosed osteoarthritis among aged over 50 years, 2010-2013 [file epih-45-e2023044-Supplementary-2.docx]

**Supplementary Material 2.** Association between breastfeeding duration with radiologically diagnosed osteoarthritis among aged over 50 years, 2010-2013

| Duration | Unadjusted | Model 1 | Model 2 | Model 3 |
| --- | --- | --- | --- | --- |
| None | 1 | 1 | 1 | 1 |
| Any duration | 2.84(1.86, 4.33) | 1.69(1.09, 2.62) | 2.45(1.50, 4.03) | 1.79(1.09, 2.95) |
|  |  |  |  |  |
| None | 1 | 1 | 1 | 1 |
| 1-6month | 1.00(0.54, 1.84) | 1.14(0.62, 2.11) | 2.17(1.09, 4.35) | 2.12(1.04, 4.31) |
| 7-24month | 1.33(0.83, 2.12) | 1.36(0.85, 2.16) | 2.36(1.35, 4.12) | 1.94(1.10, 3.40) |
| ≥25 month | 4.04(2.64, 6.19) | 1.97(1.25, 3.08) | 2.47(1.50, 4.06) | 1.76(1.06, 2.91) |
| *P* for trend | <0.0001 | <0.0001 | <0.0001 | <0.0001 |

N=5,663, OR: odds ratio, 95% CI: 95% confidence interval.

Model 1 adjusted for age.

Model 2 adjusted for age, body mass index, smoking status, drinking experience, physical activity, diabetes, hypertension, use of oral contraceptives, menopause status, total number of children breastfed, parity.

Model 3 adjusted for age, income, education level, occupation, body mass index, smoking status, drinking experience, physical activity, diabetes, hypertension, use of oral contraceptives, menopause status, total number of children breastfed, parity.
