# Supplementary material for: Associations of breastfeeding duration and the total number of children breastfed with self-reported osteoarthritis in Korea women 50 years and older: a cross-sectional study
Source: Epidemiol Health. 2023 Apr 13;45:e2023044. doi: 10.4178/epih.e2023044 (PMC10396802; doi:10.4178/epih.e2023044)
Supplement: Supplementary Material 3 — Association between total number of children breastfed with radiologically diagnosed osteoarthritis among aged over 50 years, 2010-2013 [file epih-45-e2023044-Supplementary-3.docx]

**Supplementary Material 3.** Association between total number of children breastfed with radiologically diagnosed osteoarthritis among aged over 50 years, 2010-2013

| Total number of  children breastfed | Unadjusted | Model 1 | Model 2 | Model 3 |
| --- | --- | --- | --- | --- |
| None | 1 | 1 | 1 | 1 |
| 1~2 | 1.35(0.86, 2.13) | 1.33(0.85, 2.09) | 1.26(0.81, 1.98) | 1.13(0.71, 1.79) |
| 3~4 | 3.32(2.14, 5.17) | 1.95(1.24, 3.09) | 1.75(1.11, 2.77) | 1.36(0.86, 2.16) |
| 5 | 7.75(4.99, 12.0) | 2.61(1.62, 4.20) | 2.45(1.50, 4.03) | 1.79(1.08, 2.95) |
| *P* for trend | <0.0001 | <0.0001 | <0.0001 | <0.0001 |

N=5,663, OR: odds ratio, 95% CI: 95% confidence interval.

Model 1 adjusted for age.

Model 2 adjusted for age, body mass index, smoking status, drinking experience, physical activity, diabetes, hypertension, use of oral contraceptives, menopause status, parity.

Model 3 adjusted for age, income, education level, occupation, body mass index, smoking status, drinking experience, physical activity, diabetes, hypertension, use of oral contraceptives, menopause status, parity.
