# Supplementary material for: Associations of breastfeeding duration and the total number of children breastfed with self-reported osteoarthritis in Korea women 50 years and older: a cross-sectional study
Source: Epidemiol Health. 2023 Apr 13;45:e2023044. doi: 10.4178/epih.e2023044 (PMC10396802; doi:10.4178/epih.e2023044)
Supplement: Supplementary Material 4 — Association between breastfeeding duration and with radiologically diagnosed osteoarthritis stratified by age group, 2010-2013 [file epih-45-e2023044-Supplementary-4.docx]

**Supplementary Material 4** Association between breastfeeding duration and with radiologically diagnosed osteoarthritis stratified by age group, 2010-2013

| Duration | Age group | | |  |
| --- | --- | --- | --- | --- |
|  | 50-59years(N=2,297) | 60-69 years(N=1,852) | Over70 years(N=1,514) |  |
| None | 1 | 1 | 1 |  |
| Any duration | 1.14(0.31, 4.21) | 2.89(1.23, 6.76) | 1.47(0.58, 3.74) |  |
|  |  |  |  |  |
| None | 1 | 1 | 1 |  |
| 1-6month | 2.04(0.45, 9.21) | 1.20(0.30, 4.7863) | 2.50(0.54, 11.64) |  |
| 7-24 month | 1.20(0.30, 4.86) | 2.89(1.12, 7.45) | 1.82(0.61, 5.39) |  |
| ≥25 month | 1.09(0.30, 4.03) | 2.94(1.25, 6.91) | 1.46(0.57, 3.71) |  |
| *P* for trend | <0.0001 | <0.0001 | <0.0001 |  |

N=5,663, OR: odds ratio, 95% CI: 95% confidence interval.

^1^ Adjusted for income, education level, occupation, body mass index, smoking status, drinking experience, physical activity, diabetes, hypertension, use of oral contraceptives, menopause status, total number of children breastfed, parity.
