# Supplementary material for: Associations of breastfeeding duration and the total number of children breastfed with self-reported osteoarthritis in Korea women 50 years and older: a cross-sectional study
Source: Epidemiol Health. 2023 Apr 13;45:e2023044. doi: 10.4178/epih.e2023044 (PMC10396802; doi:10.4178/epih.e2023044)
Supplement: Supplementary Material 5 — Association between total number of children breastfed with radiologically diagnosed osteoarthritis stratified by age group, 2010-2013 [file epih-45-e2023044-Supplementary-5.docx]

**Supplementary Material 5.** Association between total number of children breastfed with radiologically diagnosed osteoarthritis stratified by age group, 2010-2013

| Total number of  children breastfed ^1^ | Age group | | |
| --- | --- | --- | --- |
|  | 50-59years(N=2,297) | 60-69 years(N=1,852) | Over70 years(N=1,514) |
| None | 1 | 1 | 1 |
| 1~2 | 1.01(0.48, 2.14) | 1.54(0.72, 3.30) | 0.85(0.32, 2.25) |
| 3~4 | 1.45(0.61, 3.45) | 1.70(0.81, 3.55) | 1.02(0.41, 2.54) |
| 5 | 1.14(0.31, 4.21) | 2.89(1.23, 6.76) | 1.47(0.58, 3.74) |
| *P* for trend | <0.0001 | <0.0001 | <0.0001 |

N=5,663, OR: odds ratio, 95% CI: 95% confidence interval.

^1^ Adjusted for income, education level, occupation, body mass index, smoking status, drinking experience, physical activity, diabetes, hypertension, use of oral contraceptives, menopause status, parity.
